# Critically Appraised Topic on Low-Level Laser Therapy (LLLT) in Dogs: An Advisable Treatment for Skin Diseases?

**DOI:** 10.3390/vetsci9090505

**Published:** 2022-09-14

**Authors:** Roberta Perego, Martina Mazzeo, Eva Spada, Daniela Proverbio

**Affiliations:** Department of Veterinary Medicine and Animal Sciences (DIVAS), University of Milan, 26900 Lodi, Italy

**Keywords:** photobiomodulation, low-level laser therapy, fluorescence biomodulation, photodynamic therapy, therapeutic laser, dermatology, dogs

## Abstract

**Simple Summary:**

Low-level laser therapy (LLLT) is a therapeutic technique with reported regenerative, anti-inflammatory, antibacterial, and analgesic effects. In the last few years, LLLT has been used in dogs for the management of different skin lesions and diseases. This study reports a literature review using the critically appraised topic (CAT) method to determine the canine skin diseases for which LLLT is an advisable treatment. Only primary clinical prospective studies were considered. A meticulous literature search revealed 19 significant clinical trials, and these were critically analyzed. The evaluation of the best accessible evidence in July 2022 suggests that LLLT can be a promising and effective adjunctive treatment in combination with systemic antibiotic therapy for canine interdigital pyoderma and canine deep pyoderma. Furthermore, the use of LLLT is not recommended as a therapy for pedal pruritus secondary to canine atopic dermatitis. In other canine skin diseases, there is a possible helpful effect of LLLT; however, the evidence for its use is not currently convincing.

**Abstract:**

Low-level laser therapy (LLLT) is a therapeutic option that stimulates cellular function through intracellular photobiological and photochemical reactions, promoting better tissue repair and an anti-inflammatory, antibacterial, and analgesic effect. Previous studies in human and veterinary medicine have shown the clinical efficacy of LLLT in many fields. In this study, the literature was reviewed using the critically appraised topic (CAT) method to determine the canine skin diseases for which LLLT is an advisable treatment. A meticulous literature search revealed 19 significant clinical trials, which were critically analyzed. The evaluation of the best accessible evidence in July 2022 suggests that fluorescence biomodulation (FBM), a type of LLLT, can, in combination with systemic antibiotic therapy, be a promising and effective adjunctive treatment for canine interdigital pyoderma and canine deep pyoderma. Furthermore, the evidence suggests that the use of LLLT is not recommended as a therapy for pedal pruritus secondary to canine atopic dermatitis. For other canine skin diseases included in the CAT, although LLLT appears to be a promising treatment, there is not yet good scientific evidence to recommend its use.

## 1. Introduction

Low-level laser therapy (LLLT) is a noninvasive, easy-to-apply therapeutic option, with minimal side-effects. It uses photons at diverse wavelengths via a nonthermal mechanism to affect biological activity [1]. The use of LLLT is increasing in human and veterinary medicine; LLLT has been studied in a number of species, and a variety of clinical uses in veterinary medicine have recently been reviewed [2,3,4]. However, to date the precise biochemical mechanism of LLLT is not totally understood [1]. LLLT does not exploit thermal or ablative mechanisms but instead stimulates cellular function. The photons emitted by the laser or LED are absorbed by the mitochondrial chromophores (particularly cytochrome c-oxidase) or by the chromophores contained in the photoconverting substrate (applied prior to exposure to the light source), stimulating oxidative phosphorylation to increase ATP production and reduce oxidative stress [5,6]. These effects improve tissue repair, while having anti-inflammatory, anti-edema, antibacterial, and analgesic activity [7]. Different techniques are included under the term LLLT, such as photobiomodulation (PBM), fluorescence biomodulation (FBM), and photodynamic therapy (PDT). PBM involves the application of a coherent (laser) or noncoherent (filtered lamps or light-emitting diode (LED)) light source with a wavelength between 600 and 900/1200 nm [1]. A variety of substrates have been used to create the lasers used for PBM [8]. FBM involves direct application to the lesions to be treated of a photoconverting gel holding chromophores, which is subsequently illuminated by a blue LED lamp and, thus, generates fluorescence, which acts on the target tissues [2]. PDT consists of the application of a photosensitizing agent (such as aminolaevulinic acid, the precursor of porphyrins) to the pathological tissue, followed by illumination with light with a wavelength in the visible spectrum. The ensuing oxygen-dependent reaction generates reactive oxygen species (ROS) which have cytotoxic or immunomodulatory results [9]. Considering the widespread use of LLLT in veterinary medicine in the last two decades, particularly in the dermatological field, it would be useful to know the skin diseases in dog for which LLLT is really an advisable therapy.

## 2. Materials and Methods

*Clinical scenario*: The owner of a 4 year old Labrador Retriever dog affected by canine atopic dermatitis, suffering from recurrent otitis, pododermatitis, and pedal pruritus, asks the veterinarian for information about LLLT for dermatological diseases. This is a therapy which he has read about on the internet.

*Structured question:* In which canine skin diseases is LLLT an advisable therapy with good scientific evidence?

*Search strategy*: The PUBMED, Google Scholar, Web of Science (Science Citation Index Expanded), Agricola and CAB Abstract databases were examined during July 2022 using the following string: (photobiomodulation OR fluorescence biomodulation OR therapy therapeutic laser OR low level laser therapy OR low level light therapy OR low light therapy OR photodynamic therapy OR fluorescent light) and (dog OR dogs OR canine) and (veterinary) and (skin OR dermatology OR dermatological). Filters were set relating to the language used (English) and the publication period (from 1999 onward). Congress proceedings, book chapters, and reviews were excluded, and only prospective clinical studies in dogs were considered.

## 3. Results

The literature search in PUBMED identified 19 articles consistent with the desired characteristics, relevant to the clinical question and compliant with the inclusion criteria in the study. Other databases, such as Google Scholar, Web of Science, CAB Abstract, and Agricola were also searched, but no other relevant articles were found in addition to those previously identified on PUBMED. The selected articles, all written in English, consisted of prospective clinical studies, published from 1999 to 2022 (Table 1).

RCT: randomized controlled trial; PBM: photobiomodulation, FBM: fluorescence biomodulation; PDT: photodynamic therapy; SPMW: simultaneous superpulsed and multiple wavelengths.These studies met the inclusion criteria and addressed the clinical question but had very dissimilar study designs. The scientific quality of each study was analyzed employing the following parameters to establish the risk-of-bias assessment of treatment efficacy [29], as summarized in Table 2:

- *Levels of evidence*: assigned according to the previously identified criteria for therapeutic studies [30,31]. Briefly, level IA = systematic review (with homogeneity) of randomized control trials (RCTs); level IB = individual RCT (with narrow confidence intervals); level IC = all or none study; level IIA = systematic review (with homogeneity) of cohort studies; level IIB = individual cohort study; level 2C = “outcomes” ecological studies; level IIIA = systematic review (with homogeneity) of case–control studies; level IIIB = individual case–control study; level IV = case series (or poor-quality cohort and case–control study); level V = expert opinion without explicit critical appraisal or based on physiology bench research or “first principles” [31].
- *Randomization*: presence of a method of randomization and hiding of the allocation of subjects to the intervention groups to the people recruiting the participants. Score: 0 (no)–1 (yes).
- *Blinding*: trial investigator(s) blinded to the treatment allocation. Score: 0 (no)–1 (yes).
- *Similarity between groups*: populations allocated to different groups in the trial share the same characteristics at the start and throughout the study. Score: 0 (no), 1 (deduced from the text), 2 (yes).
- *Equal treatment of groups*: populations allocated to different groups in the trial treated similarly except for the therapy. Score: 0 (no), 1 (deduced from the text), 2 (yes).
- *Presence of at least 12 months follow-up*: score: 0 (no)–1 (yes).
- *Group size*: score: 0 (<10 subjects), 1 (10–20 subjects), 2 (21–40 subjects), 3 (>40 subjects).

As described in Table 2, at the end of this step of quality assessment, each study was graded [32] according to total score: conclusive evidence (total score 8–10) [18,21,23], highly suggestive evidence (total score 6–7) [13,16,17,20,22], suggestive evidence (total score 4–5) [11,14,15,19,26], or inconclusive evidence (total score ≤ 3) [10,12,24,25,27,28]. Studies showing inconclusive evidence were excluded from further assessment. 

To emphasize the overall strength of chosen studies, we evaluated conclusive, highly suggestive, and suggestive studies for each dermatological pathology treated (Table 3), considering the following variables:

- *Type of laser used*: diode laser, dual diode laser, solid medium diode laser, or LED lamp with photoconverter gel, with indication of wavelength, energy density, and power, when reported.
- *Treatment frequency (posology)*: twice a day, daily, every other day, twice a week, or weekly administration.
- *Study limits reported in the article*: presence/absence.
- *Ease of administration:* yes: good acceptance of therapy in most subjects; no: difficult to administer, annoying, or painful.
- *Adverse effects*: score 0 = none; score 1 = yes, mild or rare (<10%); score 2 = yes, moderate or common (≥10%); score 3 = yes, common and moderate or severe.
- *Number of administrations carried out*: score 1 = ≤5 administrations; score 2 = 6–10 administrations; score 3 = >10 administrations.
- *Efficacy of the treatment*: WCG = only treatment group without control group; NS = no statistical difference between treatment group and control group; SD = statistical difference between treatment and control group.

All studies showed a score of 0 (none) for adverse effects; hence, this category is omitted from Table 3.

**Table 2:** Assessment of scientific evidence of the studies. The studies are categorized as conclusive evidence (total score 8–10), highly suggestive evidence (total score 6–7), suggestive evidence (total score 4–5), or inconclusive evidence (total score ≤ 3).

Year	Reference	Level of Evidence	Randomization	Blinding	Group Size	Similarity between Groups	Equal Treatment of Groups	Follow-Up (>12 Months)	Total Score	Grading of Evidence
1999	Lucroy et al. [10]	IV	0	0	0	0	0	0	0	Inconclusive
2014	Stich et al. [18]	IB	1	1	2	2	2	0	8	Conclusive
2014	Olivieri et al. [27]	IV	0	0	0	1	1	0	2	Inconclusive
2015	Kurach et al. 2015 [13]	IB	1	1	1	2	2	0	7	Highly suggestive
2016	Perego et al. [20]	IB	1	1	0	2	2	0	6	Highly suggestive
2016	Perego et al. [14]	IB	1	0	0	1	2	0	4	Suggestive
2018	Gammel et al. [15]	IB	1	1	1	0	2	0	5	Suggestive
2019	Wardlaw et al. [16]	IB	1	1	1	2	1	0	6	Highly suggestive
2019	Sellera et al. [25]	IV	0	0	0	0	0	0	0	Inconclusive
2019	Salvaggio et al. [17]	IB	1	1	1	1	2	0	6	Highly suggestive
2019	Marchegiani et al. [21]	IB	1	1	2	2	2	0	8	Conclusive
2020	Tambella et al. [26]	IB	1	0	3	0	1	0	5	Suggestive
2020	Marchegiani et al. [12]	IV	0	0	0	0	0	0	0	Inconclusive
2020	Apostopoulos et al. [28]	IV	0	0	0	0	0	0	0	Inconclusive
2020	Marchegiani et al. [24]	IV	0	0	0	0	0	0	0	Inconclusive
2021	Schnedeker et al. [19]	IB	1	1	1	0	2	0	5	Suggestive
2021	Hoisang et al. [11]	IB	1	0	2	0	2	0	5	Suggestive
2021	Marchegiani et al. [23]	IB	1	1	2	2	2	0	8	Conclusive
2022	Marchegiani et al. [22]	IV	0	1	1	2	2	0	6	Highly suggestive

IB: randomized controlled trial, IV: poor-quality case–control study or cases series. *Randomization* score: 0 (no)–1 (yes); *blinding* score: 0 (no)–1 (yes); *group size* score: 0 (<10 subjects), 1 (10–20 subjects), 2 (21–40 subjects), 3 (>40 subjects); *similarity between groups* score: 0 (no), 1 (deduced from the text), 2 (yes); *equal treatment of groups* score: 0 (no), 1 (deduced from the text), 2 (yes); *follow-up (*>12 *months)* score: 0 (no)–1 (yes). Articles with conclusive evidence grading are shown in bold.

**Table 3:** Overall strength of LLLT in different dermatological conditions. Only conclusive, highly suggestive, and suggestive studies are analyzed.

Ref.	Disease Treated	Type of Laser Used	Treatment Frequency	Easy to Use	Number of Administrations Score	Reported Study Limits	Efficacy Score
[18]	**Pedal pruritus in AD**	**Solid medium diode laser, 12 W (maximum power) laser with dual-wavelength output 980 nm (80%) and 810 nm (20%), power 4.5 mW**	**30 s EOD for the 2 weeks, then twice a week for 2 weeks**	**Yes**	**2**	**Application protocol derived from human studies and independent of the hair length/type; absence of untreated group; possible placebo or systemic effect on the untreated paw**	**NS**
[20]	Pododermatitis	Solid medium diode laser, wavelength 808 nm, power 250 mW, energy density 0.9 J/min/cm^2^	1.5 + 6 min on the first day, then 6 min daily for 5 days	Yes	1	Small sample, one treatment per day	SD
[21]	**Interdigital pyoderma**	**LED lamp with photoconverter gel, peak wavelength between 440 and 460 nm, energy density between 55 and 129 mW/cm^2^**	**2 min, twice weekly until clinical resolution**	**Yes**	**3**	**Pruritus not evaluated as response to therapy**	**SD**
[22]	Interdigital furuncolosis	LED lamp with photoconverter gel, peak wavelength between 440 and 460 nm, energy density between 55 and 129 mW/cm^2^	2 + 2 min with 1 min rest between one illumination and the other, once weekly until clinical resolution	Yes	3	Not reported	SD
[23]	**Deep pyoderma**	**LED lamp with photoconverter gel, peak wavelength between 440 and 460 nm, energy density between 55 and 129 mW/cm^2^**	**2 min, twice weekly until clinical resolution**	**Yes**	**3**	**Pruritus not evaluated as response to therapy**	**SD**
[13]	Wound	Dual diode laser (7.5 mW/diode), wavelength 635 nm, total energy density 1.125 J/cm^2^	5 min EOD until complete reepithelization	Yes	3	Use of historical control group	NS
[14]	Wound	Solid medium diode laser, wavelength 808 nm, power 250 mW, energy density 0.9 J/min/cm^2^	6 min twice daily for 5 days	Yes	2	Possible spillover effect; no clinical healing follow-up	NS
[15]	Wound	Type of laser not reported, wavelength 980 nm, energy density 5 J/cm^2^, power 2–3.5 W	1.33–2.00 min daily for 5 days	Yes	1	Small sample; no medical history; no conclusion for traumatic, chronic, infected, or delayed healing wound; failure to rule out systemic effect of LLLT; open wound assessed only subjectively	NS
[16]	Wound	Diode laser, wavelength 850 nm, energy density 8 J/cm^2^	Min not reported; daily for 7 days	Yes	2	Small sample; no recorded time of treatment for each patient; inability to draw conclusion about many risk factors due to abnormal wound healing	SD
[17]	Wound	LED lamp with photoconverter gel, wavelength between 440 and 460 nm, energy density between 55 and 129 mW/cm^2^	2 min for 5 times until 13th day	Yes	1	Small sample; no conclusion for traumatic, chronic, infected wound	SD
[11]	Wound	Group L1: diode laser 830 nm, 4 J/cm^2^, 200 mW Group L2: diode laser 830 nm, 4 J/cm^2^, 200 mW + super pulsed diode laser 100 mW of 660 nm, 250 mW of 850 nm, and 50 mW of 905 nm	Group L1: 3.45–41.40 min EOD for 2 weeks Group L2: 3.45–41.40 min EOD for 2 weeks + 1 min/4 cm^2^ wound area one time	Yes	Group L1: 2 Group L2: 2 + 1	Small sample size; different wound size; variability of subjects; absence of histopathological analysis; small aperture of probe	SD
[26]	Otitis externa	LED lamp with photoconverter gel, wavelength between 440 and 460 nm	30 s soft + 1 min high power Group QW: weekly for 6 weeks Group BW: twice weekly for 6 weeks	Yes	Group QW: 2 Group BW: 3	Different OTIS-3 score between group at D0; clinical assessment not blinded; intact tympanic membrane for inclusion	SD
[19]	Acral dermatitis	Diode laser, wavelength between 470 and 640, average power 130 mW with a dose of 15.6 J and fluence of 3.93 J/cm^2^	2 min EOD for 2 weeks, then twice weekly for 2 weeks	Yes	2	Small sample size; empirical selection of dose and frequency of laser administration	NS

Ref: reference; AD: atopic dermatitis; EOD: every other day. *Number of administrations* score 1 = ≤5 administrations, score 2 = 6–10 administrations, score 3 = >10 administrations; *efficacy of the treatment* score WCG = only treatment group without control group, NS = no statistical difference between treatment group and control group, SD = statistical difference between treatment and control group. Articles with conclusive results are shown in bold.

## 4. Discussion

Despite the variety of published articles, in the international literature, there are few prospective clinical studies conducted in vivo that provide good scientific evidence to evaluate the effectiveness of LLLT as a treatment for canine skin diseases.

On the basis of the results obtained from this CAT, only three of the 19 studies were considered conclusive. The study of Marchegiani et al. [21] on canine interdigital pyoderma is one of the three, with a total score of 8, characterized by good methodological quality and scientific evidence. For this reason, FBM can be recommended for the therapy of interdigital pyoderma in dogs. This prospective randomized blinded clinical study on 36 dogs evaluated the effect of a LED lamp with a photoconverter gel system, used for 2 min twice weekly until clinical resolution, in combination with systemic antibiotics on clinical manifestations of canine interdigital pyoderma. This was compared to dogs treated with antibiotics alone as control group. A statistically significant decrease was noted in measured parameters for the treatment group compared to the control group. The mean time to lesion resolution was 4.3 weeks in treatment group and 10.4 weeks in control group. 

The second study [23] was performed by the same authors and with the same experimental design, but on 35 dogs affected by deep pyoderma, regardless of body location. The total score was 8, and the study was characterized by good methodological quality and scientific evidence. It was a prospective randomized blinded clinical trial evaluating the effect of a LED lamp with a photoconverter gel system, used for 2 min twice weekly until clinical resolution in combination with systemic antibiotics on clinical manifestations of canine deep pyoderma. This was compared to control dogs treated with antibiotics alone. A statistically significant decrease was recorded in the measured parameters for the treatment group compared to the control group. The mean time to resolution of lesions was 5.7 weeks in the treatment group and 11.7 weeks in the control group. For this reason, FBM can be recommended for the therapy of deep pyoderma in dogs.

It is interesting to note that, in both these trials, the duration of the course of systemic antibiotic therapy was significantly reduced if FBM was administered as an additional treatment. 

The study by Stich at al. [18] is the third study with good methodological quality and scientific evidence that was rated with “conclusive evidence”, with a total score of 8. This study demonstrated that the use of PBM is not beneficial as a treatment for pedal pruritus secondary to canine atopic dermatitis. This was a prospective, randomized, double- blinded, intraindividual study (with each dog serving as their own placebo control) on 30 client-owned dogs with symmetrical pedal pruritus secondary to canine atopic dermatitis. PBM was not effective as a localized treatment; only 38% of patients treated with PBM had a reduction of more than 50% in pruritus or lesion scores for the treated paw compared to baseline values, and there was no significant difference in scores between the paws of individual dogs treated with PBM and placebo laser. Scores decreased significantly for untreated paws, as well as in the treatment group, and the authors postulated that this improvement probably represented a placebo effect (the principal investigator and the owners were informed that one paw was being treated with PBM), although it is also possible that PBM caused a systemic effect for both treated and untreated paws in the same subject [18]. 

With regard to the other canine skin diseases investigated in this critically appraised topic, all LLLT methods were promising in many of the included studies. In fact, in most of the articles analyzed, LLLT led to an improvement in symptoms of treated subjects and often to their complete resolution [10,11,12,16,17,20,22,24,25,26,27,28]. In four studies (three in surgical or surgically created wounds [13,14,15] and one [19] in acral lick dermatitis), the LLLT did not show any significant clinical efficacy in the treated subjects. However, all clinical studies, unlike the studies by Stich et al. [18] and Marchegiani et al. [21,23], were characterized by insufficient scientific evidence for an incomplete and inappropriate methodology, as listed below. Indeed, it is important to distinguish the clinical outcome of treated subjects with the scientific-based evidence of a study, which is the purpose of a CAT. In the field of LLLT used on dogs with skin diseases, many of the studies published were unfortunately clinical cases series, thus strongly limiting their scientific evidence.

It is not possible to conclusively recommend or not recommended the use of LLLT for management of surgical wounds and incisions, because the relevant studies [13,14,15,16,17], although all randomized and blinded studies, were rated only with “highly suggestive or suggestive evidence” mainly due to the small number of subjects treated and the lack of adequate follow-up. For acute traumatic and chronic wounds, two studies [10,12] were not randomized or blinded and, therefore, categorized with “inconclusive evidence”, while another study [11], randomized but not blinded, was rated as “suggestive evidence”.

In the case of otitis externa, one study, not blinded or randomized, was categorized with “inconclusive evidence” [25], while the other, randomized but not blinded, had “suggestive evidence” [26]. For acral lick dermatitis [19] and sterile pyogranulomatous pododermatitis [20], the studies were categorized with “highly suggestive or suggestive evidence”, while, for perianal fistulas, the study was graded as “inconclusive evidence”, due to the lack of a control group [24]. In noninflammatory alopecia, the study was graded as “inconclusive evidence”, due to a lack of randomization and blinding [27]; for bacterial skin infection associated with calcinosis cutis [28], the study was rated as “inconclusive evidence” due to the lack of a control group, randomization, and blindness.

A separate case is the very recent study by Marchegiani et al. [22], an update on FBM for the treatment of interdigital furunculosis. The trial tested the once-weekly administration on 12 dogs affected by interdigital pyoderma by comparing the results obtained with those of a previous study of 2019 [21] with identical structure (inclusion/exclusion criteria, blinding scheme, scoring system, etc.) but twice-weekly administration. Unlike the 2019 study, which was “conclusive”, this new study was categorized only as “highly suggestive”, due to the lack of randomization and the fewer subjects treated. This, therefore, suggests that once-weekly administration of FBM for interdigital furunculosis cannot be recommended at the moment.

In all evaluated studies, PBM, FBM, and PDT proved to be noninvasive, easy to deliver in unsedated dogs, and without side-effects during the investigation period.

Further research in this field is indicated to increase our understanding of this new therapeutic option in the veterinary dermatological field. Generalizable, in vivo, randomized, double-blind, and controlled studies are required on a sufficiently large scale.

The subject inclusion criteria, the group randomization process, and the blinding procedure should always be described in detail in future studies. Moreover, to allow the comparison of results, all new studies evaluating the efficacy of LLLT as a treatment for canine dermatological diseases should follow the same clinical criteria for the inclusion of subjects, use the same clinical score when monitoring healing, and follow up subjects after treatment for at least 12 months, so as to be able to identify any relapses.

Through further studies, it would also be interesting to investigate the potential of LLLT to reduce the use of antibiotics, a potential advantage that emerged from the studies by Marchegiani et al. [21,23], in which duration of systemic antibiotics was reduced following concurrent FBM. The antimicrobial effect of LLLT has not yet been totally proven, although some human in vitro dentistry studies have highlighted this aspect [33,34]. The use of antibiotics is a very hotly debated topic with regard to the development of antimicrobial resistance, which is considered a global public health crisis that threatens our ability to successfully treat bacterial infections [35].

Further studies are also required to draft standardized protocols, which are currently inadequate, relating to the optimal parameters of the therapeutic lasers to be used and the posology for the treatment of each pathology. Factors such as spot size, wavelength, energy density, power density, pulse structure, total energy, total power, delivery mode (contact, point, and wide beam), the duration of the treatment, and the treatment intervals might influence the success of the LLLT. It is evident that, to obtain positive results with LLLT, each of these dosimetric parameters must be controlled within a limited range of values [36].

## 5. Conclusions

In this critically appraised topic on the use of LLLT as a treatment for canine skin diseases, good scientific evidence was identified only for the recommendation of fluorescence biomodulation (FBM) for management of canine interdigital pyoderma and canine deep pyoderma, in combination with systemic antibiotic therapy.

LLLT has the potential to be a promising treatment for many canine skin diseases. However, additional valid and generalizable clinical studies, with good scientific evidence, are required to investigate its actual efficacy and potential antimicrobic effect, as well as to produce scientifically validated standardized therapeutic protocols.

## Figures and Tables

**Table 1:** Articles included in the critically appraised topic.

Ref.	Disease Treated	Type of Therapy	Authors and Year	Study Population and Study Design	Interventions and Outcomes	Results
[10]	Chronic wounds	PBM	Lucroy et al., 1999	Single case report of an 8 year old castrated Whippet dog	Irradiation on the awake dog with 630 nM wavelength once daily for 4 successive days. Changes in wound surface zone calculated by computer analysis of digital images of the wound.	The wound diminished in size during the trial and was completely healed by day 21. No post-treatment complications occurred.
[11]	Chronic wounds	PBM	Hoisang et al., 2021	RCT of 21 owned dogs of different breeds, genders, and ages	Dogs assigned into three groups: (1) control group (C) managed with irrigated saline and without PBM (*n* = 7); (2) L1 group with irrigated saline together with PBM radiation at 830 nm (*n* = 7); (3) L2 group with irrigated saline together with SPMW-PBM radiation (*n* = 7). Wound healing estimated on wound size decrease as a percentage of wound zone every 2nd day for 15 days employing image analysis software.	A significant difference in the percentage of wound area reduction was recorded between the C and PBM groups at the end of the study (15 days). A consistent decrease in wound size was observed in both PBM and non-PBM groups. The percentage of wound area reduction was significantly different between the PBM and non-PBM groups on day 7 (*p* < 0.05).
[12]	Acute traumatic wounds	FBM	Marchegiani et al., 2020	Two case reports of two aged mixed-breed dogs	FBM therapy began 5 days after the initial presentation in both dogs. The wound was then covered with a bandage to avoid contamination. The whole process was duplicated once a week until wound healing.	Wound closure and wound healing were fulfilled after 9 and 16 weekly treatments, respectively, with a total re-epithelization of the skin. The small degree of wound contraction did not restrict the free movements and apparently did not disturb the dogs (no signs of suffering or tendency to self-trauma).
[13]	Bilateral trunk wounds, surgically created	PBM	Kurach et al., 2015	RCT of 10 adult (13–18 months of age), purpose-bred, male Beagle dogs	Each side randomized to get LLLT or standard-of-care management 3 times weekly for 32 days. Wound planimetry carried out on the caudal wounds, from which percentage contraction and percent epithelialization were estimated. Histologic features were assessed at 7 timepoints from cranial wound biopsies. Obtained data were also correlated to wounds from a female control cohort of a previous study.	No difference between treated and control wounds for all parameters, as well as in terms of histology. Gender may impact wound healing in intact dogs.
[14]	Post-neutering surgical skin wounds of at least 3 cm in length	PBM	Perego et al., 2016	RCT of seven client-owned dogs of different age and breed that underwent ovariectomy for elective sterilization	One-half of the wound randomly selected and managed with LLLT and the other left untreated. The protocol was twice daily, 6 min, laser treatments for 5 days. The treated and control areas assessed with a clinical score on the first day (D0) and at the end of laser treatment (D4).	Almost all treated areas had considerable and visible clinical improvement compared to control areas. Nevertheless, statistical analysis revealed that there was no significant difference between the two groups at D4. No adverse reactions were reported.
[15]	Surgically closed incisions and surgically created open wounds	PBM	Gammel et al., 2018	RCT of 10 dogs of different breeds that underwent bilateral flank ovariectomy procedures, aged 6 months to 5 years	Dogs were subjected to bilateral flank ovariectomy procedures and open wounds generated bilaterally with a punch biopsy. Each side of the dog (open wound and incision) was randomly allocated to the treatment group or the control group. The treatment group received LLLT once daily for 5 days with a 980 nm laser. The control group received a fake treatment (laser turned off) for an equal amount of time each day. Wounds evaluated visually and biopsied on postoperative days 7 and 14.	There was no difference between groups for subjective evaluation of healing time and wound measurements. There was no difference in histopathologic evaluation except that the control group at day 7 had more necrosis and perivascular lymphocytes and macrophages.
[16]	Surgical wounds	PBM	Wardlaw et al., 2019	RCT of 12 Dachshund dogs that underwent thoraco-lumbar hemilaminectomies for intervertebral disc disease	First three dogs utilized to develop a standardized scar scale to score the other dogs’ incision healing. The other 9 dogs randomly allocated to either receive laser therapy once a day for 7 days or the control group (untreated). Incision healing scored using a scar scale from 0 to 5, with 0 denoting a fresh incision and 5 denoting completely healed with scar contraction and hair growth. Photographs were collected within 24 h of surgery and 1, 3, 5, 7, and 21 days postoperatively.	All scar scores significantly improved with extending time from surgery. Good inter-rater reliability. Laser therapy increased the scar scale score and revealed improved cosmetic healing, by day 7 to day 21, compared to control dogs.
[17]	Surgical wounds	FBM	Salvaggio et al., 2019	RCT of 10 healthy client-owned dogs of different genders, ages, and breeds that underwent orthopedic surgery	Half of the length of each surgical wound randomly assigned to treatment with FBM, and the remaining 50% was handled with saline solution on the first day after surgery and every 3 days until day 13. Wound healing of treated and control areas evaluated with macroscopic assessment and histological and immunohistochemical analysis. The surgeon and the pathologists were blind to the treatment designations.	The FBM areas treated reached lower histology scores, with complete re-epithelialization, less inflammation of the dermal layer, and bigger and more regular collagen deposition. As revealed by immunohistochemistry, expression of factor VIII, decorin, collagen III, epidural growth factor, and Ki67 raised in treated compared with untreated tissues.
[18]	Symmetrical pedal pruritus	PBM	Stich et al., 2014	RCT of 30 client-owned dogs which satisfied at least six of the seven diagnostic criteria for atopic dermatitis	Dogs randomly assigned to one of two study groups. Group A: LLLT on the right paw and placebo on the right paw; Group B: placebo on the left paw and LLLT on the left paw. The principal investigator and owners were unaware of the group designations. Each dog experienced three laser sessions per week over the course of weeks 1 and 2, two laser sessions per week in weeks 3 and 4, and no laser treatments in week 5. At weeks 0, 2, 4, and 5, dogs assigned a score (localized canine atopic dermatitis severity score—LCADSS) by the principal investigator and a score (localized pruritic visual analog score—LPVAS) by the owner, and cytology assessed. The primary outcome assessment was a >50% reduction from baseline of the LCADSS and LPVAS.	There were no significant dissimilarities in LCADSS or LPVAS between LLLT and placebo treatments between weeks 0 and 5. However, LCADSS and LPVAS significantly decreased from week 0 at weeks 2, 4, and 5 in both LLLT and placebo groups.
[19]	Acral lick dermatitis	PBM	Schnedeker et al., 2021	RCT of 13 owned dogs of different breeds, genders, and ages	Dogs were treated with systemic antibiotics and trazodone and randomly allocated to two groups. The treatment group (TG) received LLLT by laser (130 mW, 2 min) with blue and red light-emitting diodes (LEDs), while the control group (CG) had fake therapy (laser/LEDs off). Treatments managed three times weekly for 2 weeks, then twice weekly for 2 weeks for a total of 10 times. The licking visual analog scale (LVAS) was developed. The LVAS questionnaire was completed by the owner at each visit until study end, and the scores were registered by the unblinded examiner.	There were no significant dissimilarities in median LVAS, lesion/ulcer size, or thickness of the lesion between TG and CG. There was a significantly bigger increase (24%) in hair growth in TG compared to CG.
[20]	Multiple lesions of sterile pyogranulomatous pododermatitis	PBM	Perego et al., 2016	RCT of five client-owned dogs of different genders, ages, and breeds	One lesion randomly allocated as control (treated with a 0.0584% hydrocortisone aceponate spray), and one or more other lesions managed with LLLT daily for 5 days. Lesions clinically scored before treatment (D0), at the end (D4), 16 days after the last laser treatment (D20), and after 2 months (D65).	There was a statistically significant difference at D4 and D20 between treated and control groups; in the treated group over time, there was a statistically significant advancement between D0, D4, and D20. Lesion recurrence was not present in more than 50% of the treated lesions at D65. No adverse reactions were recorded.
[21]	Interdigital pyoderma	FBM	Marchegiani et al., 2019	RCT of 36 privately owned dogs of different genders, ages, and breeds	Dogs randomly and blindly assigned to treatment groups of either antibiotic alone (control group) or antibiotic and twice-weekly FBM treatment (FBM group). Dogs scored over a 12 weeks period on the basis of two evaluated parameters: a global lesion score comprised of four different lesions types and neutrophil engulfing bacterial scores.	A statistically significant decrease recorded by week 3 in both measured parameters for FBM group compared to control group. The mean time-to-resolution of lesions was 4.3 weeks in FBM group and 10.4 weeks in control group.
[22]	Interdigital pyoderma	FBM	Marchegiani et al., 2022	Cases series of 12 privately owned dogs of different genders, ages, and breeds	Dogs received antibiotic and once weekly FBM. Dog scores compared with the results obtained from twice-weekly FBM application in a previous study with same inclusion/exclusion criteria, blinding scheme, scoring method, and clinical evaluation [21]	Once-weekly FBM application exerts the same beneficial effect of interdigital foruncolosis healing as twice weekly.
[23]	Deep pyoderma	FBM	Marchegiani et al., 2021	RCT of 35 privately owned dogs of different genders, ages, and breeds	Dogs randomly and blindly assigned to treatment groups of either antibiotic alone (control group) or antibiotic and twice-weekly FBM application (FBM group). Assessments began weekly for 8 weeks and every 2 weeks thereafter until 12 weeks after admission.	After 8 weeks of treatment, the percentage of dogs that reached clinical resolution was 35.0% and 88.0% for control group and FBM group, respectively. Lesions cores showed highly statistically significant difference in favor of FBM group from week 3 to 8, and neutrophil engulfing bacteria scores showed statistical difference from week 2 forward in favor of FBM group.
[24]	Perianal fistulas	FBM	Marchegiani et al., 2020	Case series of four owned dogs of different genders and ages	FBM used as only therapy once a week with two consecutive applications in the same session for each dog until clinical signs had significantly improved, with weekly evaluations for a 6 week period. Dogs were evaluated by measuring the size of lesions at the start of the study and then weekly for 6 weeks, using software. Owners recorded vocalization and distress frequency scores during their pet’s defecation, as well as perianal licking frequency on a 0–5 point scale to evaluate the response to therapy.	All dogs got better with FBM, achieving a significant reduction in vocalization, straining, and licking after 2 weeks. After 5 weeks of therapy, lesional areas had significantly decreased. Only one dog required more than seven treatments. No adverse events were noted.
[25]	Spontaneous otitis externa	PDT	Sellera et al., 2019	Single case report of a 5 year old Lhasa apso dog	Unilateral otitis externa caused by carbapenem-resistant *P. aeruginosa* treated with antimicrobial photodynamic therapy (aPDT) using methylene blue as photosensitizer. The isolated bacterial strain also checked for susceptibility to in vitro aPDT. For decolonization, probiotic supplements were orally used (once daily) for 14 days. Effectiveness of probiotics and photodynamic therapy evaluated by clinical and microbiological culture assays.	Total resolution of clinical signs reached by day 7 after aPDT. Samples obtained immediately and after 7 and 14 days following aPDT negative for VIM-2-producing *P. aeruginosa*. Oral and rectal swabs obtained on days 7, 14, and 21 after probiotic therapy validated effective gastrointestinal decolonization.
[26]	Spontaneous otitis externa	FBM	Tambella et al., 2020	RCT of 37 owned dogs of different genders, ages, and breeds	Dogs randomly assigned to three groups: group QW with a topical LED-illuminated gel (LIG) once weekly; group BW with LIG twice weekly; group C with enrofloxacin and silver sulfadiazine twice daily. The estimation protocol (T0 to T5) considered clinical assessment (OTIS-3 index scoring system; pruritus severity scale; pain severity score; aural temperature), cytological scoring system, and quali-quantitative bacteriologic evaluation.	All groups achieved improvement during the study. The greatest clinical score reduction appaired in Group BW. BW obtained a clinically relevant effect level at T3, QW reached it at T4, and C did not reach it. No differences between groups were noted in the reduction in CFU/mL (T0–T5).
[27]	Noninflammatory alopecia	PBM	Olivieri et al., 2014	Case series of seven privately owned dogs of different ages, genders, and breeds	Unsedated dog treated twice weekly for a maximum of 2 months with therapeutic laser with three different wavelengths. A fixed alopecic area left untreated and used as a control area. The efficacy assessed clinically by visual examination. Areas documented and with photographs and graded at the start of the study, after eight applications (4 weeks) and at the end of the study. From one dog, post-treatment biopsies of treated and untreated sites achieved for histological determination of hair density and the percentage of haired and non-haired follicles.	At the end of the study, coat regrowth greatly improved in 6/7 animals and improved in 1/7. Via morphometry, the area occupied by hair follicles was 18% in the treated sample and 11% in the untreated one (11%); haired follicles were (per area) 93% in the treated sample and only 9% in the control sample.
[28]	Calcinosis cutis with secondary pyoderma	FBM	Apostolopoulos et al., 2020	Single case report of a 15 year old male Golden Retriever dog	FBM used as an auxiliary to systemic antimicrobial and topical therapies. Part of the lesions covered with a towel and not exposed to FBM, to determine clinical and cytological efficacy.	Cytology supported increased improvement of the illuminated lesions compared with unexposed lesions.

## Data Availability

All data discussed are contained in the manuscript.
